# Transparent superwetting nanofilms with enhanced durability at model physiological condition

**DOI:** 10.1038/srep19178

**Published:** 2016-01-14

**Authors:** Sunghee Hwangbo, Jiwoong Heo, Xiangde Lin, Moonhyun Choi, Jinkee Hong

**Affiliations:** 1 School of Chemical Engineering and Material Science, Chung-Ang University, Seoul 156-756, Korea

## Abstract

There have been many studies on superwetting surfaces owing to the variety of their potential applications. There are some drawbacks to developing these films for biomedical applications, such as the fragility of the microscopic roughness feature that is vital to ensure superwettability. But, there are still only a few studies that have shown an enhanced durability of nanoscale superwetting films at certain extreme environment. In this study, we fabricated intrinsically stable superwetting films using the organosilicate based layer-by-layer (LbL) self-assembly method in order to control nano-sized roughness of the multilayer structures. In order to develop mechanically and chemically robust surfaces, we successfully introduced polymeric silsesquioxane as a building block for LbL assembly with desired fashion. Even in the case that the superhydrophobic outer layers were damaged, the films maintained their superhydrophobicity because of the hydrophobic nature of their inner layers. As a result, we successfully fabricated superwetting nano-films and evaluated their robustness and stability.

Research on controlling the wettability of films has recently received much attention, though there are several challenges to its future progression. The superwettability, a property that ranges from superhydrophobicity (with a static contact angle, SCA > 150°) to superhydrophilicity (SCA < 5°), of thin films has been extensively investigated, due in large part to their diverse applications including biomedical devices. In particular, a superhydrophobic film could be employed in self-cleaning windows^1^,^2^, corrosion resistant barriers^3^,^4^, drag-reducing films^5^,^6^, anti-fogging devices^7^,^8^, anti-bacterial films^9^, cell-capturing films^10^,^11^,^12^ and so on.

The morphology and surface chemistry of a film are the key factors that can be controlled in order to produce superwetting characteristics^13^,^14^. Surfaces that are only covered with low surface energy materials exhibit a maximum contact angle of approximately 120°^15^. The appropriative morphology for a superhydrophobic film is that of a rough structure on both microscale and nanoscale, a two tier roughness. Also a porous structure that exhibits a high roughness is typical of superhydrophilic films. Special structures for use in superwetting films could be prepared with the help of methods such as lithography^16^ etching^17^, and particle-based film fabrication^18^. Many previous studies selecting various material combinations and conditions, such as molecular weight, particle size, number of bilayers, pH, and salt concentration, a film with a specific morphology can ultimately be fabricated^19^,^20^,^21^. By modifying the surface chemistry of a textured surface, the superhydrophobic or superhydrophilic properties of a film can be readily controlled^22^,^23^,^24^.

However, superwetting films prepared by controlling surface chemistry and morphology still present some drawbacks for commercial uses especially in nanoscale modification on devices. Their mechanical stability is poor, due either to fragility of the rough structure at micro-scale^25^ or weak coherence which caused by among the un-crosslinked polymer components of the film^26^. Once layers that have been subject to adjustments in their surface texture or chemistry become damaged by mechanical abrasion or heat, their superwetting characteristics reduce accordingly.

Due to their fragile structure, many researchers have focused on enhancing the durability of superwetting films. M. Manca *et al*. presented durable superhydrophobic and highly transparent films using trimethylsilane, which contained functionalized silica nanoparticles embedded in an organosilicate binder matrix^26^. These maintained their superhydrophobicity, with a small decrease in the contact angle, during 2,000 h of outdoor exposure. Y. C. Jung *et al*. fabricated hierarchical surfaces using a carbon nanotube (CNT) coating on a micro-pillar structure, which exhibited a high durability at water pressures of 10 kPa for 24 h^27^. Gong. G *et al*. reported the fabrication of superhydrophobic films that consisted of silica nanoparticles embedded in an electrospun polyimide matrix, giving both thermal stability and abrasion resistance^28^. In addition, T. S. Kustandi *et al*. generated nanoimprinted polymer structures that exhibited a reversible shape recovery by using a shape memory thermoplastic polymer (Nafion). These showed an ability to recover their microstructure at 140 °C^29^. These studies attempted to remedy issues related to the superwetting surfaces of films with thicknesses in the micrometer range. However, due to the trend of the gradual miniaturization of biomedical devices from micron to nanoscale, it is necessary to control the wetting properties of films with thicknesses in the nanometer range. Also, superwetting films employed in biomedical application are needed special properties such as *in vivo* stability of the constituents of a film and film with bottom up strategy for fabricating lithography free textured surfaces, regardless of the type of substrate used.

In this study, we fabricated superwetting nano-films by organosilicate-based layer-by-layer (LbL) self-assembly methods due to control their nanoscale film roughness and take full advantage of LbL assembly^20^,^30^,^31^. Using this approach, we easily fabricated nano-sized roughness structures on fragile substrates, for use in biomedical surfaces such as implantable devices^32^, contact lenses^33^, and sticking plasters^34^. Also, we introduce bio-stable materials for use as a building block for LbL assembly. The stability of nanofilms is naturally much lower than for films with a microscale thickness. For this reason, we employed polymeric newly designed silsesquioxane, prepared using the sol-gel chemistry method, in order to enhance the stability of the structure as well as endow intrinsic hydrophobicity. The main advantages of using polymeric silsesquioxane are that it readily promotes both a high stability and the multifunctionality of the film. The silsesquioxane molecular structure typically consists of a strong siloxane bond (Si-O), and therefore exhibits a strong physical and thermal stability, and chemical resistance^35^,^36^. In addition, multifunctional silsesquioxane can be synthesized with the addition of different silane coupling agents. Silane coupling agents play a critical role in our technique, by forming covalent bonds between the organic functional group and the inorganic silsesquioxane. As a result, the silsesquioxane itself can exhibit a distinct multifunctionality including surface energy by fluorinated silane^37^. In addition, polymer-nanoparticle cross-linked network could be remarkably helpful in both reducing the external force acting on the film, compared to a particle-particle structure, as the polymer layer in an LbL film serves as buffer and increasing roughness for additional hydrophobicity^38^.

In this manuscript, we synthesized fluorinate silsesquioxane (F-SQ) with an acid catalyst using tetraethoxysilane (TEOS), silane coupling agent, and perfluorodecyltriethoxysilane (PFAS) in order to prepare intrinsically hydrophobic polymeric silsesquioxane. The stability of the film was considerably increased through the addition of F-SQ, which also served to make the film intrinsically hydrophobic. To fabricate superhydrophobic surfaces, controlling morphology of film is also important. According to this selection strategy, the method for fabricating a multilayer with controllable wetting properties is briefly described as the following. First, we synthesized a mixture of F-SQ and silica nanoparticles (F-SiSQ). The mixture exhibited superhydrophilic characteristics due to their abundant hydroxyl groups (the fabrication process is shown in Fig. S1, Supporting information). BPEI (Branched polyethyleneimine) containing several hydrophilic amine groups was used as an appropriate building block for the LbL film. Because of rich hydrophilic functional groups, the synthesized (BPEI/F-SiSQ)_n_ film (BF film) showed superhydrophilic characteristics. Under the influence of the silica nanoparticles in F-SiSQ, the roughness of the BF film could be governed at the nanoscale, and they displayed the nano structure that is highly suitable in a superwetting film. In order to build up the superhydrophobic surface, the BF layer was coated with an FDTS (1 H, 1 H, 2 H, and 2 H-Perfluorodecyltrichlorosilane) monolayer that caused a low surface energy, using self-assembled monolayer methods and then heat-treated, in order to induce a condensation reaction between the film components forming the strong covalent bonds that enhanced the stability of the film. The as-prepared (BPEI/F-SiSQ)_n_ FDTS coated and annealed (referred to as the BFFA film) multilayer exhibited superhydrophobicity (SCA > 170°). In addition, not only the surface of the BFFA film but also the inner layer of the film (the (BPEI/F-SiSQ)_n_ annealed film is referred to as the BFA film) was intrinsically hydrophobic (SCA > 130°), a result of eliminated hydroxyl groups in the BFFA or BFA film and the increased effect of the presence of fluoroalkyl chains in the F-SQ. All the film fabrication processes, together with the naming scheme used, are detailed in Fig. 1. The resulting films were able to exhibit both controlled superhydrophobic or superhydrophilic properties and high stability on a nanotextured film fabricated using LbL assembly. Moreover, in terms of the effect of fluoroalkyl chains^39^ and silsesquioxane^35^, they also showed high transmittance in the visible light range. It was extremely challenging to produce a film of nanoscale thickness that exhibited superwetting characteristics and stability via a self-assembly based method, and these films can be applied in large-area processing varing from centimeter- to meter-scale. Above all, considering the high stability of these films, despite of their nanoscale thickness, the results of this work indicate that they could be suitable for biomedical applications.

## Results and Discussions

### Growth of superwetting films

BF film assembly was driven by the hydrogen bonding force (amine groups in the BPEI and hydroxyl groups in the F-SiSQ worked as the hydrogen bonding acceptor and the hydrogen bonding donor, respectively)^40^.

**Figure 2:**
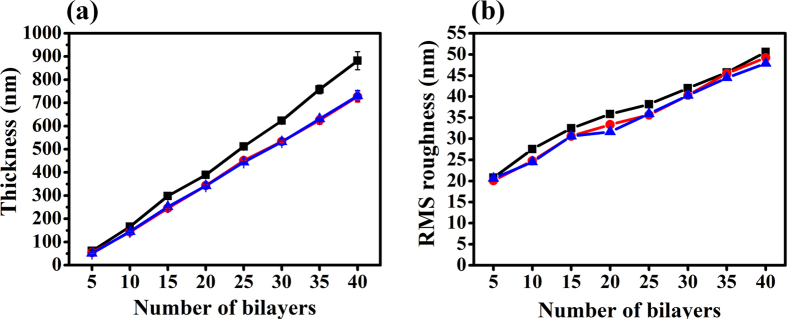
The thickness and RMS roughness of superwetting films, as a function of number of bilayers, are shown in (a,b), respectively, for BF (■, black line), BFA (•, red line), and BFFA (▲, blue line) films.

Figure 2(a,b) depicts the thickness growth and roughness profile of the LbL assembled BF film and the influence of post-treatment through a comparison of BF, BFA and BFFA films. First, BF film thickness and RMS roughness exhibited linear growth as function of the number of deposited bilayers. The thickness and roughness of the BF film show an average increase of 22.04 nm and 1.264 nm per bilayer, respectively. This result demonstrates the fact that a uniform silica particle monolayer is formed in every bilayer of the BF film, where the calculated radius of silica nanoparticles observed in F-SiSQ was 20 nm. In view of the uniform growth of the silica particle monolayer, RMS roughness also gives a roughly linear relationship with respect to number of bilayers. Therefore, by way of changing the number of bilayers, we could precisely adjust the thickness and roughness of the BF multilayer at the nanoscale. Second, the effect of heat treatment on thickness and roughness was also recorded. During heat processing, BFA film thickness and roughness per bilayer decreased by an average of 3.86 nm and 1.70 nm, respectively, until 40 bilayers of film had been deposited. Third, the thickness and roughness of the BFA and BFFA films were compared in order to determine the effect of the FDTS coating process. The thickness and roughness of the BFFA film was higher (0.560 nm, on average) and lower (0.452 nm on average), respectively, than for the BFA film. We observe that the influence of the FDTS monolayer coating is quite small due to its low thickness. As a result, the FDTS coating process has a negligible effect on BFA film morphology, and so the film could maintain its nano structure after post-treatments such as FDTS coating and the annealing process.

### Topography

**Figure 3:**
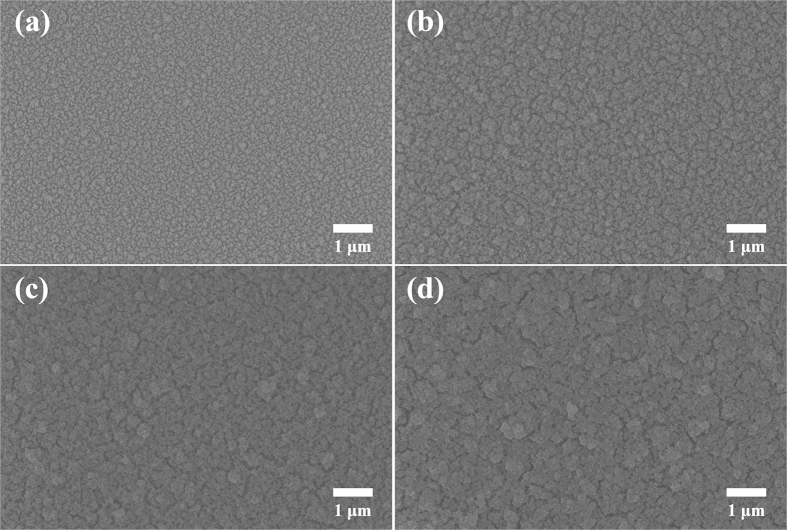
SEM top view images of BFFA films containing different amounts of bilayers. (**a**) 10 bilayers, (**b**) 20 bilayers, (**c**) 30 bilayers and (**d**) 40 bilayers.

Figure 3(a–d) shows FE-SEM top view images of BFFA films with 10, 20, 30, 40 bilayers. From this data, we can determine that there exists a micro-nanoscale roughness in the BFFA film. P. -C. Lin *et al*. studied the effect of controlled dual-scale roughness on superhydrophobic characteristics^41^. As a result, the sliding angle of surfaces with micro-nanoscale roughness decreased more than 3 times compared to those exhibiting single nanoscale roughness. Thus, dual-scale roughness is most suitable when building a superhydrophobic film. In this study, a nanoscale roughness caused by the presence of nano-sized silica particles was observed on the BF, BFA, and BFFA films, and the micro-scale roughness was a result of silica nanoparticle agglomeration. As the number of deposited bilayers increased, the agglomeration of silica nanoparticles also approached the microscale. For BFFA films containing 10, 20, 30 and 40 bilayers, the sizes of the agglomeration were on average 142.0 ± 41.6 nm, 252.77 ± 43.5 nm, 303.8 ± 35.5 nm, and 540.4 ± 56.5 nm respectively.

**Figure 4:**
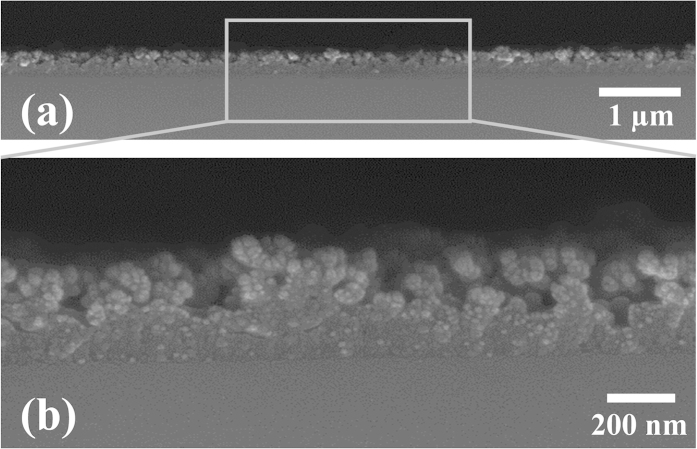
SEM cross-section images of a BFFA 20 bilayer film using different magnifications are shown in (a,b).

FE-SEM cross-section images of a BFFA film containing 20 bilayers are shown in Fig. 4. An image with increased magnification, shown in Fig. 4(b), displays the nano roughness structure of the BFFA film, together with a nanoporous structure. This nano roughness structure is particularly desirable for a superwetting film^42^,^43^, and a porous structure increases film transmittance by reducing the reflexive index of the film^44^,^45^. The low-magnification image presented in Fig. 4(a) shows a BFFA film of a homogeneous thickness, due to the LbL assembly molecular level film deposition process, while the BFFA film exhibits a particle-based rough structure.

The AFM images of the BF, BFA, BFFA films show topology changes after post-treatment (Described in Fig. S2, Supporting information). As previously mentioned, the FDTS monolayer coating and annealing processes decreased the RMS roughness of the films (BF film: 41.9 nm, BFA film: 38.5 nm, BFFA film: 37.8 nm). Any topology changes resulting from heat treatment can be determined by comparison with the BF ((a), (b)) and BFA ((c), (d)) films. The z-direction displacement of the film decreased after the annealing process but the BFA film still exhibited a nano structure similar to that of the BF film. The effect of FDTS monolayer coating can be analyzed by comparing the BFA and BFFA films ((e), (f)). As a thin, uniform coating of the FDTS monolayer was applied to the BF film, only slight morphological differences between the two films could be detected.

### Wetting properties

Several studies on superhydrophilic and superhydrophobic dual-wetting properties have previously been reported^22^,^23^,^24^ and the critical factors contributing to the superwetting characteristics of a surface are its roughness and surface chemistry. We have confirmed morphology of BF, BFA, and BFFA films. In this section, we mainly focus on the surface chemistry changes resulting from FDTS coating and annealing steps. After the impact of post-treatments, the BF, BFA, and BFFA films were superhydrophilic, intrinsically hydrophobic, and superhydrophobic, respectively. Furthermore, we will compare the surface chemistries of the BF, BFA, and BFFA films.

**Figure 5:**
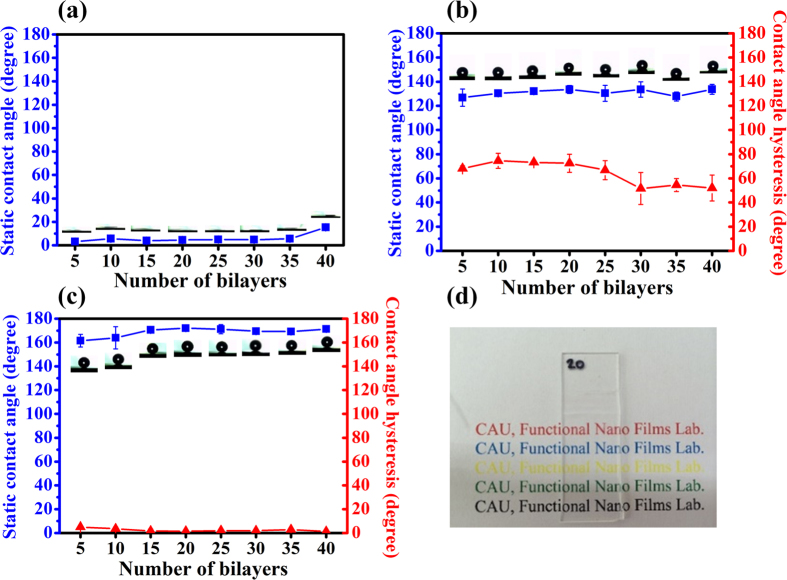
The variation of static contact angle (SCA, ■, blue line) and contact angle hysteresis (CAH, ▲, red line) values as a function of number of bilayers are shown for (a) BF films, (b) BFA films, and (c) BFFA films. An image showing a BFFA 20 bilayer film on a quartz glass substrate with high transmittance is shown in (**d**).

Due to the hydrophobicity of silsesquioxane, a small quantity of PFAS served as a coupling agent for F-SQ. The as-synthesized F-SQ therefore contained a large amount of hydrophilic-hydroxyl groups and hydrophobic-perfluoroalkyl groups. Due to the impact of long perfluoroalkyl chains, the F-SQ coated films displayed hydrophobic wetting characteristics (SCA = 110°). However in the case of F-SiSQ coated films display hydrophilic wetting property, because the majority of the silica nanoparticles coexisted with plenty of hydroxyl groups, and therefore the influence of perfluoroalkyl chains was reduced. F-SiSQ and BPEI containing hydrophilic-amine groups were used as the building blocks for the BF film, which means that the BF film was superhydrophilic, as shown in Fig. 5(a). Even though only 5 bilayers of BF film had been deposited, the average of SCA values was 3.1°. Therefore, we can readily fabricate a superhydrophilic film containing several bilayers. A superhydrophilic film can be converted into a hydrophobic surface through an annealing process. The BFA film exhibited intrinsically hydrophobic properties due to the presence of hydrophilic functional groups such as amine and hydroxyl groups, which were almost completely removed during an annealing step at a temperature of 200 °C, in vacuum conditions for 4 h. Hence, the effect and also the proportion of perfluoroalkyl chains increased, and thus the BFA film exhibited intrinsically hydrophobic characteristics. The wetting properties of BFA films as a function of number of deposited bilayers are shown in Fig. 5(b), and changes in the functional groups of both the BF film and the F-SQ were caused by the annealing process, as described in Figs S3,4, Supporting information. The average contact angle of the BFA films is presented in the range from 5 to 40 bilayers (at intervals of 5 bilayers). Similar to the case of the BF film, only a small number of BFA bilayers are needed in order to create intrinsically hydrophobic surfaces. The sample containing 5 bilayers of BFA exhibited intrinsically hydrophobic properties with an SCA of 127°, while the sample coated with 40 bilayers of BFA exhibited a maximum SCA and lowest contact angle hysteresis (CAH) value of 134° and 52°, respectively. Deviations in the SCA values of the BFA film were not large (with an average of 131 ± 3°), although the CAH values of the BFA films exhibited a trend of decreasing with increasing number of deposited bilayers, which arose from the increase in the RMS roughness.

In order to understand the intrinsically-hydrophobic characteristics of the BFA film that were caused by annealing, Fourier Transform Infrared Spectroscopy (FT-IR) was employed. FT-IR spectra of F-SQ depicts the, before ((a), a pristine sample) and after the annealing process ((b), an annealed sample), together with a table giving the corresponding peak positions. Before the annealing process, F-SQ contained a large number of hydroxyl groups, and so the silanol (Si-OH) peaks were observed at 3326 and 927 cm^−1^. However, in high-temperature conditions, almost all the hydroxyl groups in the F-SQ condensated and underwent a conversion into siloxane (Si-O-Si) groups. Therefore, B (the annealed F-SQ) sample presented a 13.38% increase in the Si-O-Si band area (asymmetric cyclic Si-O-Si: 1143 cm^−1^, linear asymmetric Si-O-Si: 1070 cm^−1^, and linear symmetric Si-O-Si : 787 cm^−1^)^46^,^47^ and a 78.00% decrease in the Si-OH bands area, which indicates that a high degree of condensation occurred during the annealing process. These phenomena show not only a decline in the amount of hydrophilic functional groups but also an increase in the coherence between heat-treated BF film components. The FT-IR spectra of the BF, BFA, and BFFA films also indicate the reinforcement of coherence between film components. In the BF film spectrum, the peaks at 2932 and 1582 cm^−1^ were in correspondence with the secondary amine in the BPEI. However, distinct changes can be observed in the BFA and BFFA films compared with the BF film. The absorption peaks of the secondary amine groups were reduced and converted into primary amine peaks (1655 cm^−1^)^48^,^49^, and these changes indicate a condensation reaction in the BFA and BFFA films. In order to fabricate superhydrophobic surfaces, an FDTS monolayer was deposited on the dual-scaled BF film using the self-assembly monolayer method to control the surface chemistry of the film. A film containing only 5 deposited bilayers gives a SCA of 162° and a CAH value of 5°, which are located in the superhydrophobic region (SCA > 150°, CAH > 10°). We noted a decrease in CAH and increase in SCA for the BFFA films as a function of the number of deposited layers. The SCA and CAH values for a BFFA film containing 40 bilayers were 171° and 1°, respectively. These phenomena indicate that the BFFA films exhibited a uniform nano structure and their surface chemistry was well controlled by the fluoroalkyl chains in the FDTS monolayer. The presence of fluoroalkyl chains in the FDTS monolayer of a BFFA film was verified using the FTIR spectra. As the FDTS monolayers were very thin (0.462 nm), the intensity of perfluoroalkyl chains was low, although we observed increments in the perfluoroalkyl chains bands, such as the rocking CF_2_ peak (647 cm^−1^) and the wagging CF_2_ peak (571 cm^−1^), in relation to the BF and BFA films. The contact angle and FTIR results clearly demonstrated that the surface chemistry and wetting properties of the BF, BFA, BFFA films differed.

### Transmittance

Figure 5(d) shows the BFFA 20 bilayer film on quartz glass. Despite its thickness of 341.16 nm, the film displayed antireflection properties (AR), together with a high transmittance. The optical transmittance of superhydrophobic BFFA films containing various numbers of deposited bilayers on quartz glass, that were measured using UV-vis spectroscopy in the visible light range is presented in Fig. S5, Supporting information. We deposited 10 and 20 bilayers of BFFA film in order to evaluate the effect of varying film thickness on film transmittance. The transmittance graphs confirm several points. First, we may note that the transmittance of BFFA films was higher than that of bare quartz glass substrates at wavelengths beyond 412 nm (10 bilayer sample) and 500 nm (20 bilayer sample). When compared with uncoated quartz, the quartz covered with a superhydrophobic BFFA film exhibited AR properties as a result of two factors. In order to fabricate films with AR properties, many reports have been focused on porous surfaces and materials with a low refractive index^50^. The first factor is the presence of F-SiSQ in the BFFA film, which has a low reflective index, and the second is related to the nanoporous structure of the BFFA film. M. Saito *et al*. reported on the refractive losses of polymethyl methacrylate (PMMA), polyimide, and fluorocarbon polymer (CYTOP)^39^. In this study, the reflective index of CYTOP was found to be 1.35 ± 0.05, while the minimum refractive index in a homogeneous dielectric material is 1.34, due to their low absorption coefficients. H. Hoerauf *et al*. also examined the refractive indices of various perfluoroalkyl-substitituded liquids, such as perfluoro-octane, perfluoro-decane, and perfluoro-hexylethane, and their refractive indices ranged from 1.27 to 1.34^51^. Low-refractive-index materials effectively reduce the amount of light loss caused by reflection. Fluoroalkyl chains are superior to the other polymers for use as an AR coating material. The nanoporous structure of a BFFA film was detailed in Fig. 4(a). There are various approaches used to fabricate nanoporous surface structures, such as the block copolymer film^52^, particle based sol-gel film^53^, and surface etching film^54^ methods, which effectively reduce the refractive index of a film. Due to the low refractive index of BFFA films, they are highly transparent and can be used as antireflection coatings.

There are distinct differences in the optical transparencies exhibited by BFFA coatings of different thicknesses. V. Kumar *et al*. investigated the optical properties of films, stating that they depend strongly on film thickness^55^. The transmittance of a film will be decreased with an increasing number of bilayers. The thicknesses of the 10 and 20 bilayer BFFA films in this study were 145.05 nm and 342.16 nm, respectively, as shown in Fig. 2(a). The optical transmittance of the (BFFA)_10_ films was almost 5.48% lower than that of the (BFFA)_20_ film, at a wavelength of 550 nm, due to its thickness and the red-shift in the absorption edge. In order to fabricate an ideal AR coating, the thickness of the coating should be equal to 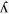/4^45^,^56^. The optical thickness of the BFFA film was 137.5 nm, almost equal to that of 8 bilayers of BFFA film. However, as discussed in Fig. 5(c), the CAH of the BFFA film finally reached the value of less than 2° when more than 15 bilayers of film were deposited. As the number of deposited bilayers was increased, the superwetting characteristics of the films were improved, but the film transmittance decreased as a result of increasing film thickness. Also, the high roughness accompanying a large number of BFFA film bilayers caused light scattering that reduced the AR properties of a film^45^. However, 20 bilayers of BFFA film with superhydrophobic properties exhibited a high transmittance in the range from 400 nm to 800 nm, together with a transmittance of approximately 99.99% at 550 nm, whereas the transmittance of untreated quartz glass at this wavelength was only approximately 96.62%. These experimental results indicate that BFFA films are a promising candidate for self-cleaning windows that require superhydrophobicity and high transmittance.

### Film stability

**Figure 6:**
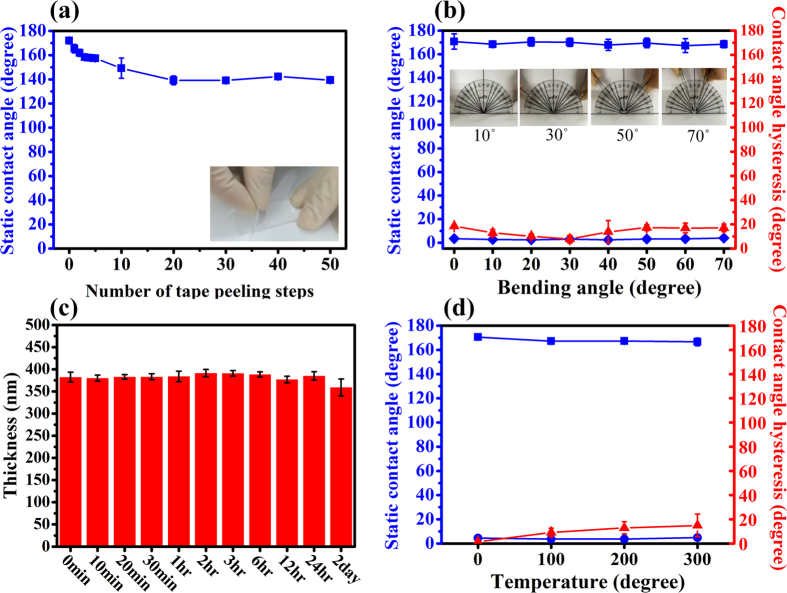
Variation of static contact angle (SCA, blue line) and contact angle hysteresis (CAH, red line) values after the durability testing of BF and BFFA films. A tape peeling test, bending test, and thermal stability test are presented in (**a,b,d**), respectively. The change in thickness of a BFFA film during 2 days of PBS stability testing is shown in (**c**).

Our proposed film fabrication method mainly employed silsesquioxane as a layer-by-layer building block because of their high stability resulting from the presence of strong siloxane bonds. The stability of our films was revealed by the presence of covalent bonding between the BPEI and the F-SiSQ, and the F-SiSQ with itself, as discussed before, by using the FTIR results. Here, the stability enhancements of superwetting films are discussed, which may be considered to be the strongest point of our research. The mechanical durability of our prepared superwetting films was tested using tape peeling, bending, and phosphate buffered saline (PBS) stability tests, and their thermal stability was verified by heat testing. A quantitative discussion of the physical strength of the films arising from the presence of silsesquioxane is also included. Figure 6(a) shows that the static contact angle for the prepared superhydrophobic surface of the BFFA film, as a function of the number of times the tape was peeled. It could resist the peeling test 50 times, with a slight decline in hydrophobicity. At first, the SCA of the BFFA film was 172°, but after peeling 20 times, the SCA was reduced to 139°, and then this contact angle was maintained up to 50 peeling times. This decrease in the contact angle at the early stages (10 bilayers) can be explained by a delamination of weakly molecular level thickness of FDTS monolayers from the surface of the BF film. During 10 peeling tests, the unbonded FDTS monolayer was partially eliminated, and only few FDTS monolayer remained on the BFFA film. There is also an intrinsically hydrophobic layer (134°) under the FDTS monolayer coating. For these reasons, the BFFA film maintains its hydrophobicity during additional peeling steps. In addition, we also attempted to estimate the stability of BF films using a peeling test. However, it was difficult to measure the changes in the contact angle due to high adhesive forces between the BF film and the scotch tape (Fig. S6, Supporting information). After one step of tape peeling test, the adhesive material of the scotch tape became strongly attached to the BF film without delamination. That indicates that the BF film strongly combines with the silicon wafer through hydrogen bonding.

The interfacial fracture toughness of superwetting films has been evaluated using a bending test. As shown in Fig. 6(b), 20 bilayer BF and BFFA films stacked on overhead projector (OHP) film were bent to various degrees (from 0° to 70°) and the bent structure was maintained for 1 min, after which the SCA and CAH values were measured. After the bending tests that performed drastic transformations, the SCA values of BFFA films were maintained at almost 170°, and the CAH of the BFFA film exhibited a slight change, but still maintained its initial value. As the BFFA and BF films were influenced by the OHP film substrate, the CAH values of the film used in the bending test were different from those of films deposited on the silicon wafer. As could be expected, the BF films maintained their superhydrophilic characteristics (SCA < 5°) after a 70° bending test. These results indicate that our films exhibited high flexibility without changes to their super-wetting properties, due to strong siloxane bonding in the F-SQ and coherence between the layer-by-layer building blocks.

An evaluation of the phosphate buffered saline (PBS) stability of BFFA films is given in Fig. 6(c). A high cohesion force between film components with high proportion of covalent bonding leads to a lower swelling ratio for a film in a good solvent which can dissolve components of film. There is a standard for calculating the degree of crosslinking within a film by determining the amount of swelling resulting from immersion for a day in a good solvent^57^. Due to nano-scale thicknesses of our superwetting films, we measured the change in film thickness instead of measuring the change of in mass or volume. Superhydrophobic and superhydrophilic (BFFA)_20_ and (BF)_20_ films were immersed in PBS solution for 1 day, and their thicknesses were measured as a function of immersion time in order to indirectly measure the degree of crosslinking. As the driving force of BF film formation is hydrogen bonding, it is the weaker BFFA film that consists of strong covalent bonds. During the PBS solution stability test, the thickness of the BF film was maintained for 2 hours, although after this point, the film thickness began to decrease. (Fig. S7, Supporting information) However, the BFFA film maintained its thickness for more than 24 hours in PBS solution (Fig. 6(c)) and its maximum variation in its thickness was only approximately 2.6%. The BF film was weaker than the BFFA film in PBS solution because the hydrogen bonds in the BF film were weaker than the covalent bonds in the BFFA film, These results indicate that the BFFA film was stable in PBS solution because it contained a number of covalent bonds.

The contact angles of heat-treated BF and BFFA films were used in order to evaluate the thermal stability of the superwetting films, as shown in Fig. 6(d). For heat treatments in the temperature range from 100 °C to 300 °C, the SCA values of the BF and BFFA films were below 5° and above 165°, respectively, which indicates that the films maintained their superwetting properties at high temperatures. As the annealing temperature increased, the CAH values of heat-treated BFFA films slightly increased (from 9.17° in 100 °C to 14.96° in 300 °C). This is due to the loss of the FDTS coating (with a boiling point of 240 °C) and BPEI (with a boiling point of 250 °C) during the heat-treatment. When the film was heat-treated at 300 °C, its SCA and CAH values were 166.60° and 14.96°, respectively, and it approached the sufficient conditions for a superhydrophobic film (SCA > 150°, CAH < 10°). These results demonstrated that the prepared superwetting films exhibited a high resistance above the boiling points of the film components. This is due to the fact that the layer-by-layer building blocks and self-assembly monolayer coatings on the BFFA film were covalently bonded, and these bonds suppressed the evaporation of FDTS and BPEI from the film during heat treatment. The silica included in the F-SiSQ also acted as a thermal enhancement compound, improving the heat resistance of the superwetting films. Images of BFFA films after heat treatment are presented in Fig. S8, Supporting information.

We improved the stability of our film through the introduction of silsesquioxane, which consists of several strong siloxane bonds. In order to verify the stability enhancement that may be attributed to silsesquioxane, we fabricated a (BPEI/Si-NP)_n_ film without F-SQ, which was subject to annealing under the same conditions as the BFA film (at 200 °C for 4 h). We then compared its hardness and Young’s modulus with those of the BFA film using nanoindentation (See Table 1). In order to facilitate precise comparison, the film thicknesses were of similar values, of approximately 600 nm. The local hardness and Young’s modulus values of the (BPEI/Si-NP)_20_ and (BFA)_35_ films are shown in Table 1. The average hardness and Young’s modulus of the BFA film were 2.9 and 1.4 times higher than the values of the (BPEI/Si-NP)_n_ film, respectively. It is clear that the hardness and Young’s modulus increased with the presence of F-SQ. There are two reasons for this increase. First, the BFA film that contained F-SQ was composed of a harder structure compared to the (BPEI/Si-NP)_n_ film, because the silica nanoparticles were well dispersed in the polymer-like F-SQ matrix. A different number of bilayers was required to reach a thickness of approximately 600 nm (BFA: 35 bilayers, (BPEI/Si-NP)_n_: 20 bilayers). The second reason is that the rigid silica core included in silsesquioxane improved the mechanical properties of the BFA film.

### Application to large-area process

**Figure 7:**
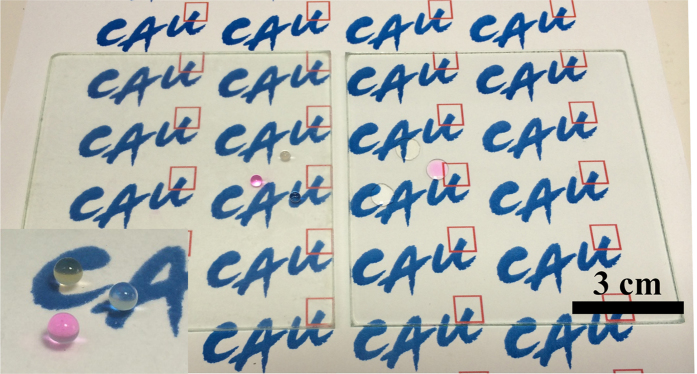
Images of a large glass slide, coated with 15 bilayers of BFFA film (left) and uncoated (right).

Textured surfaces generated by lithography, etching, and electrophoretic deposition methods are difficult to fabricate for films with a large area. In the case of particle-based layer-by-layer assembled textured surfaces, films can be generated on different types and sizes of substrates using various fabrication methods, such as dipping, spraying, spin coating, and printing. We fabricated a successfully manufactured transparent and superhydrophobic (BFFA)_20_ film on a large area (9 cm × 9 cm) of glass using the layer-by-layer dipping method (Described in Fig. 7). Taking full advantage of layer-by-layer methods, superwetting films can be employed in large-area processing for use in various commercial applications.

In summary, superwetting films were prepared by particle-based LbL assembly using silsesquioxane and silica nanoparticles in order to obtain dual-scale roughness and improved durability. In order to obtain various wetting properties, subsequent post-treatments such as annealing and FDTS coating were employed. Untreated BF films and post-treated BFFA films exhibited superhydrophilic and superhydrophobic wetting properties, respectively. Due to their nanoscale thickness and the presence of the perfluorocarbon compound in the BF and BFFA films, they exhibited a high transparency. The presence of the silica core in F-SiSQ also successfully improved the durability of the superwetting films, as was evident from the nanoindentation results. The BFFA film was more stable than the BF film, due to the condensation caused by heat treatment. There are two differences between these films that may be attributed to heat treatment: the increment of the degree of condensation present and the elimination of hydroxyl groups that induce the intrinsically hydrophobic properties of the BFA layer (the inner layer of the FDTS coating). Therefore, the components of the BFFA film are covalently bonded, and it maintains its hydrophobicity if the FDTS monolayer is eliminated. The durability of the superwetting film was verified using a peeling test, a bending test, a heat test, and a Phosphate Buffer Saline (PBS) stability test. By taking full advantage of LbL assembly, the proposed superhydrophobic and superhydrophilic films can be generated for various compositions and sizes, and easily adopted for use in various industrial applications.

## Methods

### Materials

1 H, 1 H, 2 H, and 2 H-Perfluorodecyltrichlorosilane (FDTS, MW = 581.56) was purchased from Alfa Aesar. n-Hexane was obtained from Samchen Chemical, Korea. Ethanol was provided by Daejung, Korea. The 1 H, 1 H, 2 H, 2 H-perfluorodecyltriethoxysilane (PFAS, MW = 610.38), LUDOX® TMA colloidal silica 34 wt% suspension in H2O, tetraethyl orthosilicate > 99.0% (TEOS, MW = 208.33), and branched polyethylenimine (BPEI, Mw ~25,000) were purchased from Aldrich. All chemicals were used without further purification.

### Synthesis and characterization of F-SQ and F-SiSQ

F-SQ was prepared using the following two-step process, previously reported by H. J. Jeong *et al*.^58^ First, measured amounts of TEOS (1.33 g) and PFAS (0.015 g) were added to the ethanol solution (9.56 g) for a stirring time of 20 min. Second, pH 2 water (1.14 g), as a rapid catalyst for the sol-gel reaction, was added to the resultant mixture. After a 3.5 h reaction period at room temperature in order to facilitate hydrolysis and condensation, F-SQ was prepared. Next, 1 ml Ludox TMA silica nanoparticles and 0.5 ml of F-SQ solution were added together, using a 5 min vortexing period, and then F-SiSQ was synthesized. As these steps were performed, the sol-gel reaction occurred and facilitated covalent bonding between the F-SQ and Ludox TMA silica nanoparticles.

### Layer-by-layer film fabrication procedure

A silicon oxide wafer was washed sequentially in deionized-water, acetone, and ethanol using the sonication dipping method, and then dried using N_2_ gas. The clean wafer was then treated with oxygen plasma for 2 min in order to produce a negatively charged and activated surface. The activated wafer was immersed in BPEI solution for 10 min and rinsed it thrice with ethanol for 2, 1, and 1 min. These steps were then repeated using F-SiSQ solution. Several of these cycles could be repeated in order to form the BF films. The BPEI and F-SiSQ solutions were dispersed in ethanol with a concentration of 1 mg/ml.

### Post-fabrication treatments

The BF film was treated at 200 °C, in vacuum conditions, for 4 h in order to form the BFA film through an annealing process. In order to fabricate BFFA films, the FDTS coating step was carried out by slowly dipping the samples into a solution containing 0.1 vol % of FDTS in hexane for 30 min, before withdrawing vertically at a constant speed (mm/sec). The film was then dried at room temperature in order to remove any remaining solvent. The BFFA film was annealed using the same conditions as were employed in the annealing process for the BFA film.

### Characteristics of F-SiSQ, F-SQ and multilayer films

Field-Emission Electron Microscopy (FE-SEM) images were obtained using a SIGMA electron microscope (Carl Zeiss), and Fourier Transform Infrared Spectroscopy (FT-IR) absorbance peaks were acquired using an FTIR 4700 device (JASCO). Film thickness was measured using a profilometer (Dektak 150, Veeco). The root mean square (R_rms_) roughness values and 2D, 3D AFM images were obtained with non-contact atomic force microscope (AFM, NX-10, Park systems) and hardness and young’s modulus values were obtained using Oliver and Pharr methods with diamond tip. The substrates of the films were activated using O_2_ plasma (CUTE-1B, Femtoscience). The transmittance values of the BFFA films were confirmed using a UV-visible spectrometer (V-670, JASCO). Static contact angle images were captured using a labmade contact angle goniometer, at ambient temperatures. The volume of the water droplets was 4 μL, and the contact angle of the samples was measured 5 times. A charge coupled device (CCD) camera (IMT 3, IMT solutions) was used with a contact angle goniometer and the LB-ADSA methods in free software (IMAGE J). Contact angle hysteresis experiments were conducted by using a KRUSS (model: DSA 100) goniometer by water drop volume from 4 μl to 10 μl, at a constant speed.

### Mechanical stability tests

Tape-peeling test was used to measure the surface resistance to the adhesive force of Scotch 810 tape. Tapes were attached on the multilayer films with constant finger pressure and then detached within the 2 seconds for the equal speed; this process was repeated up to 50 times. Surface resistance was investigated by measuring the SCA after every tenth peeling operation.

The multilayer films for the bending test were prepared on PDMS substrates and pressure was applied to both ends of the film by hand on the goniometer. The surface of the substrate were bent from 0°(no pressure) to 70°(maximum bent) based on the goniometer.

To verify stability under osmotic pressure, we used phosphate buffered saline (PBS), purchased from Bio solution, diluted 20 times as a model physiological environment. Multilayer films were prepared on the silicon wafer substrate that had been modified to a negative charge by the oxygen plasma. Stability of the film was observed by measuring the thickness change with a profilometer at specific time intervals for desired days. The film was stored in the physiological environment at 37 °C in an incubator during the test.

Also, we used atomic force microscope (AFM, NX-10, Park systems) with diamond nanoindentor tip (TD 23491, Park systems). The Young’s modulus and hardness of the films were analyzed by XEI software (Park systems) through force-height curves calculated by the Oliver and Pharr model.^59^

## Additional Information

**How to cite this article**: Hwangbo, S. *et al*. Transparent superwetting nanofilms with enhanced durability at model physiological condition. *Sci. Rep*. **6**, 19178; doi: 10.1038/srep19178 (2016).

## Supplementary Material

Supplementary Information

Supplementary Video S1

## Figures and Tables

**Figure 1:**
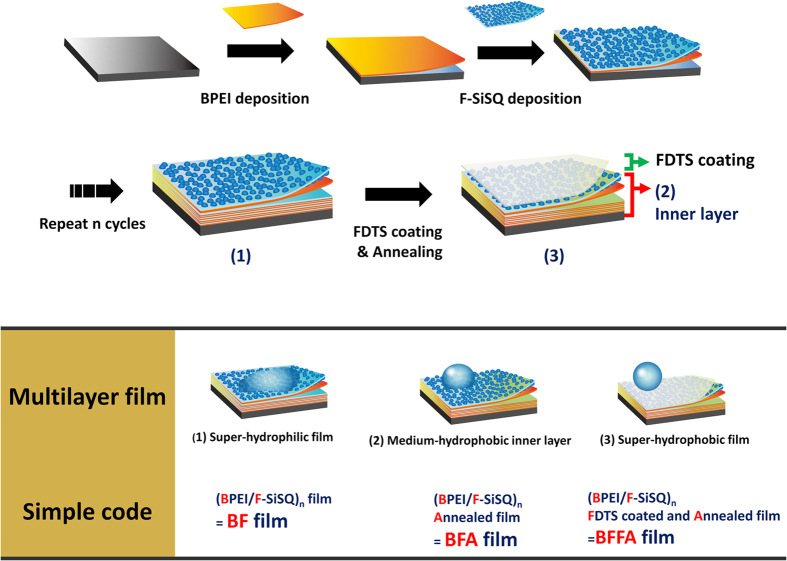
A schematic illustration of LbL deposition and the post-treatments of superwetting films, together with a description of the relevant abbreviations.

**Table 1:** Hardness and Young’s modulus of (BPEI/Si-NP)_n_ and BFA films, measured using nanoindentation.

	(BPEI/Si-NP)_20_	BFA_35_
Thickness (nm)	609.29	591.16
Hardness (MPa)	46.58	135.37
Young’s modulus (MPa)	193.64	274.90

Mechanical properties of films measured by nanoindentation.
