# Supplementary material for: Transparent superwetting nanofilms with enhanced durability at model physiological condition
Source: Sci Rep. 2016 Jan 14;6:19178. doi: 10.1038/srep19178 (PMC4725873; doi:10.1038/srep19178)
Supplement: Supplementary Information [file srep19178-s1.doc]

Supporting Information for:

Transparent superwetting nanofilms with enhanced durability at model physiological condition

Sunghee Hwangbo, Jiwoong Heo, Xiangde Lin, Moonhyun Choi and Jinkee Hong*

1. **Schematic illustrations of the fabrication procedure for F-SQ and F-SiSQ.**
2. **AFM 3D, 2D images of BF, BFA and BFFA films**
3. **Infrared spectra for F-SQ, before and after heat treatment**
4. **Infrared spectra for BF, BFA, BFFA film**
5. **Transmittance of a multilayer film**
6. **Images of BF films, before and after one step of the tape-peeling test**
7. **The thickness change of a BF film during 3 h of water stability testing**
8. **Images of BFFA films after heat treatment**
9. **Dynamic drop experiment of BFFA film**

**Figure S1. Schematic illustrations of the fabrication procedure for F-SQ and F-SiSQ.**


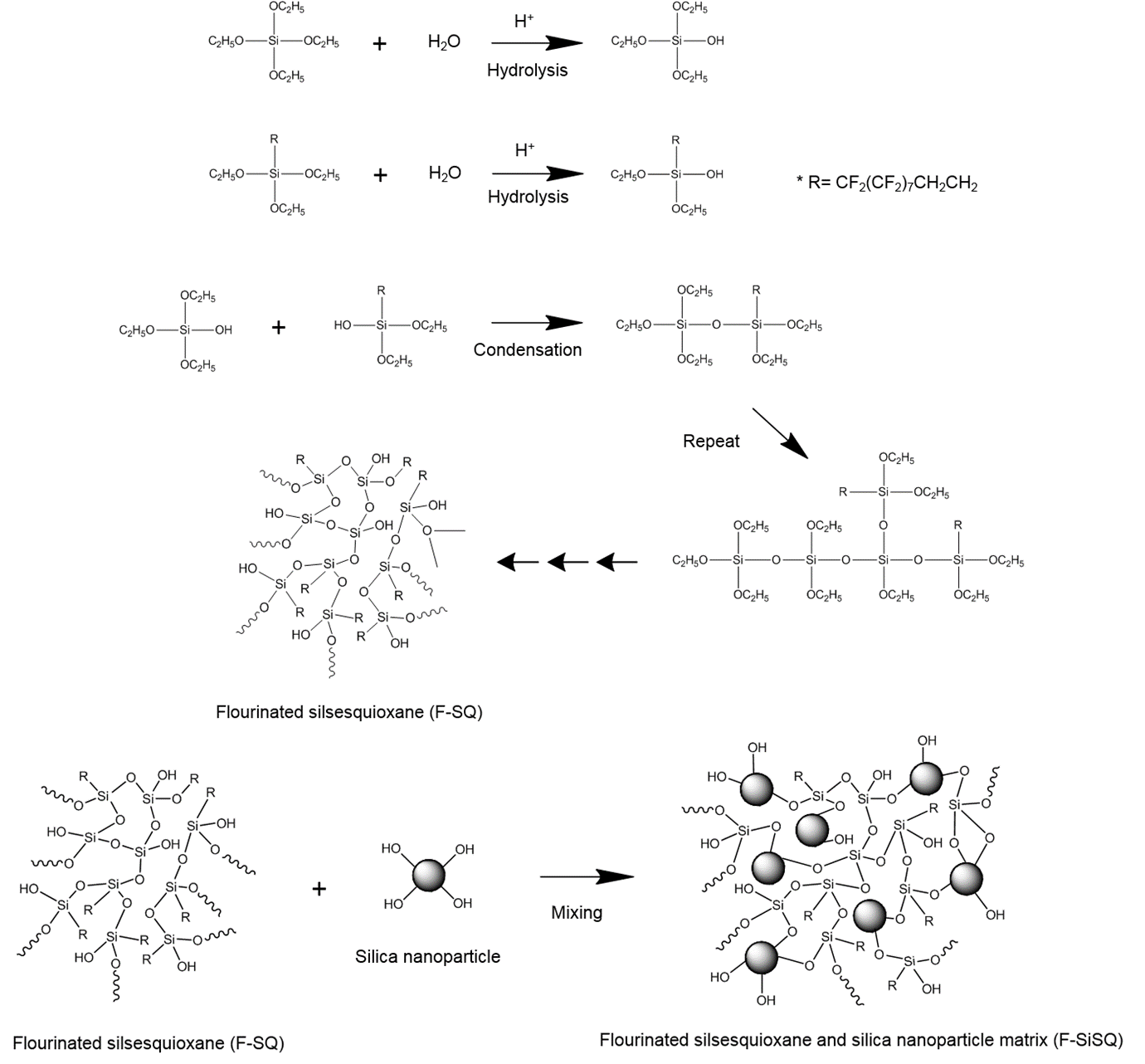


**Figure S2. The AFM images of the BF, BFA, BFFA films**


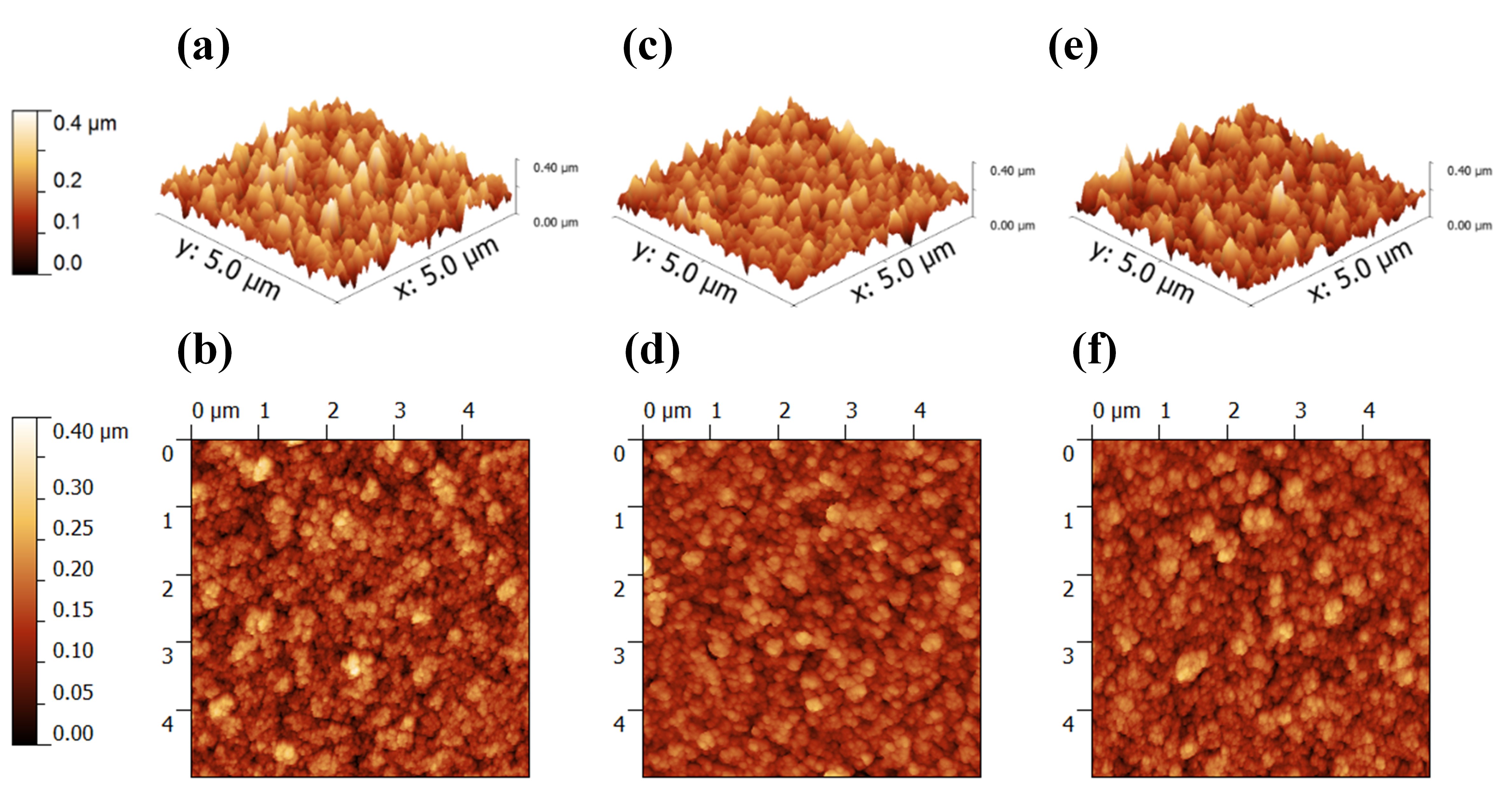
AFM 3D, 2D images of BF ((a), (b)), BFA ((c), (d)), and BFFA ((e), (f)) films.

**Figure S3. Infrared spectra for F-SQ**


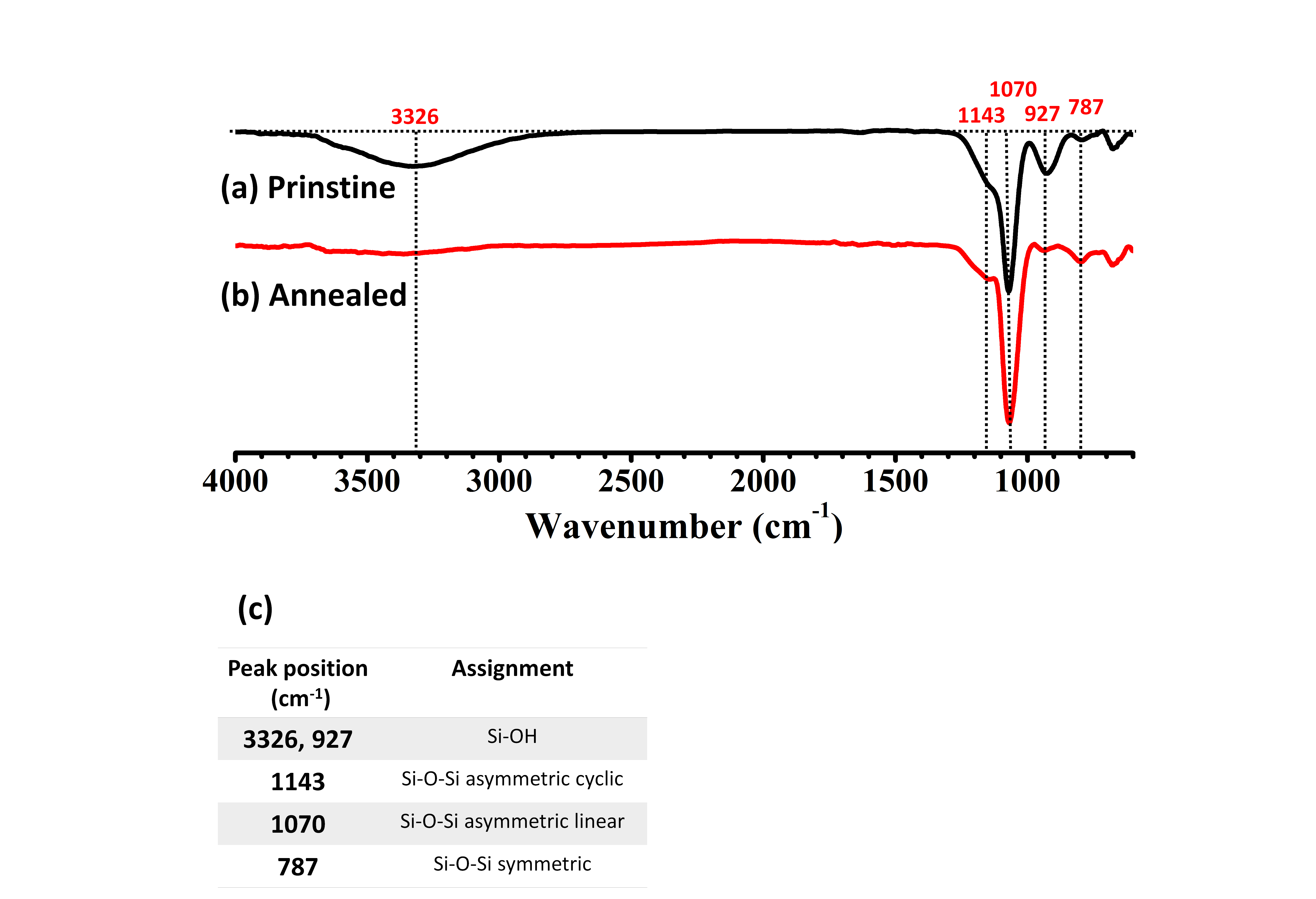


Infrared spectra for F-SQ for pristine ((a), black line) and annealed ((b), red line) spin-coated samples. The annealed sample was prepared using heat-treatment (200˚C for 4 h). Their peak positions, together with variations in the functional groups, are presented in (c).

**Figure S4. Infrared spectra for BF, BFA, BFFA films**


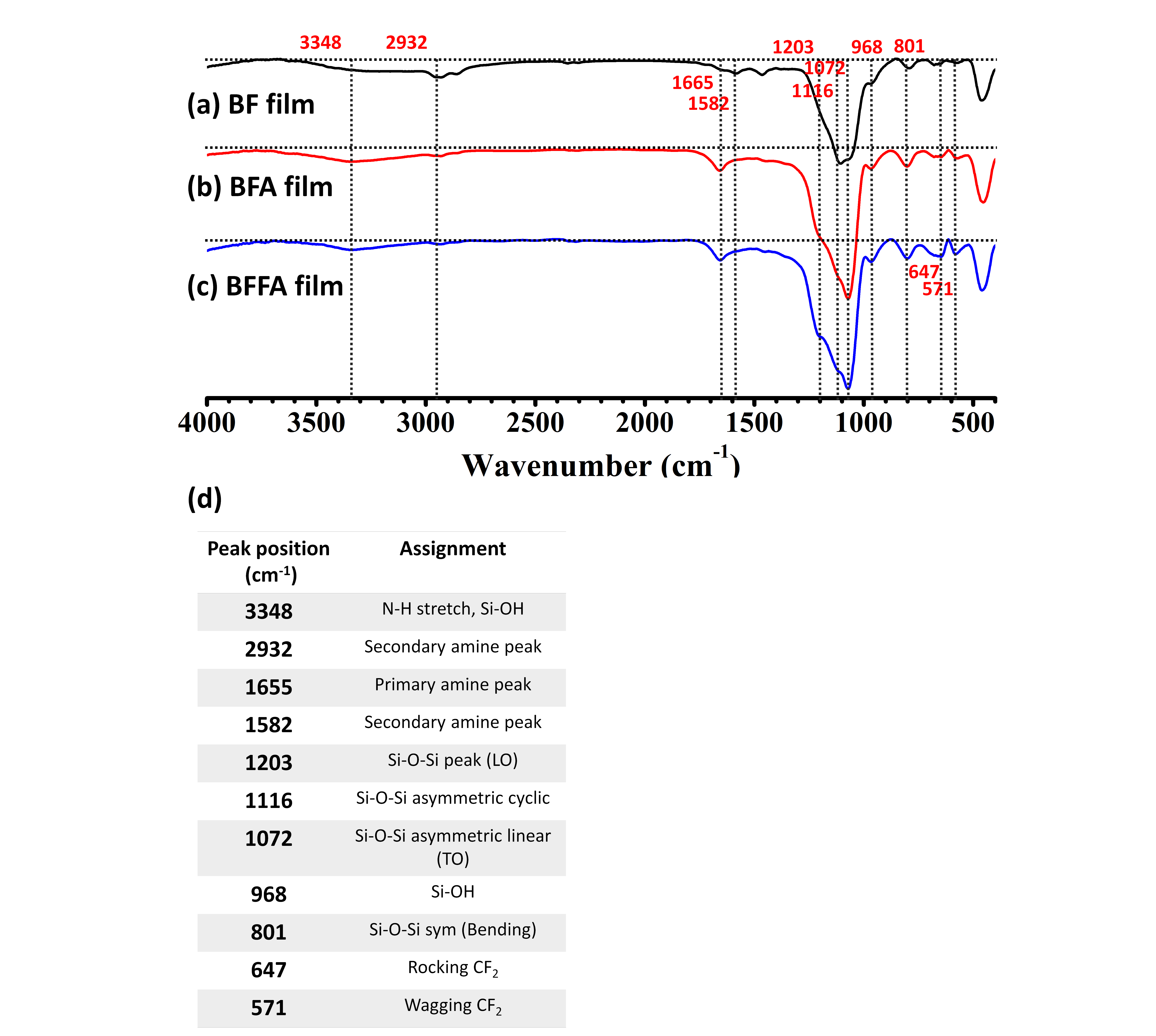


Infrared spectra of BF ((a), black line), BFA ((b), red line), and BFFA ((c), blue line) films. Their peak positions, together with variations in the functional groups, are presented in (d).

**Figure S5. Transmittance of a multilayer film**

**
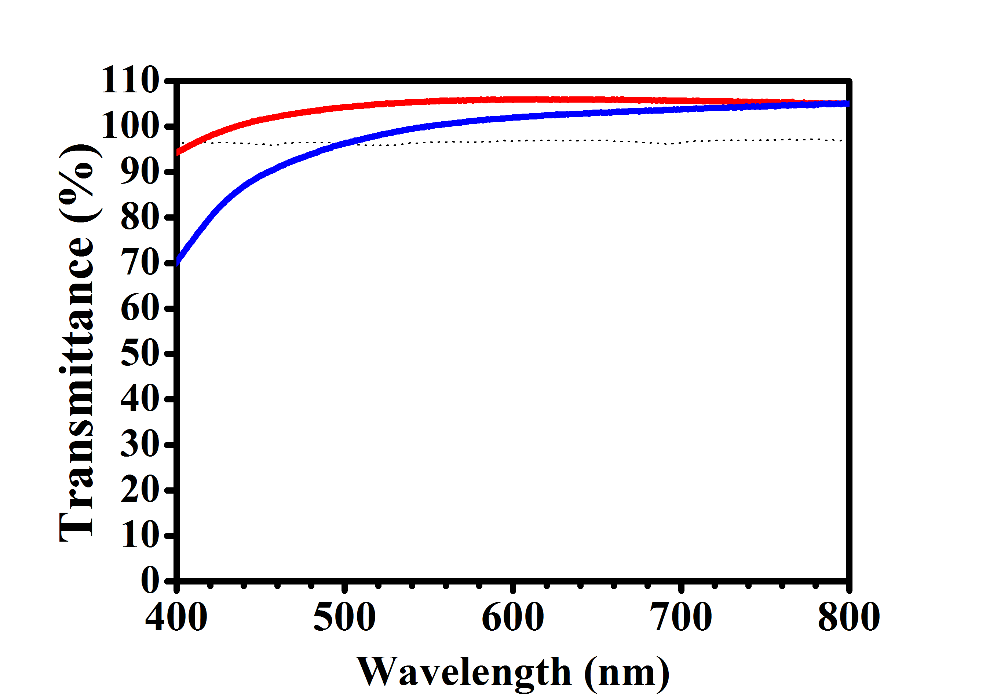
**

Transmittance of a multilayer film containing 10 (red line) and 20 bilayers (blue line) of BFFA film. The transmittance of a quartz glass sample (dotted line) is also given

.

**Figure S6. Images of BF films, before and after one step of the tape-peeling test**


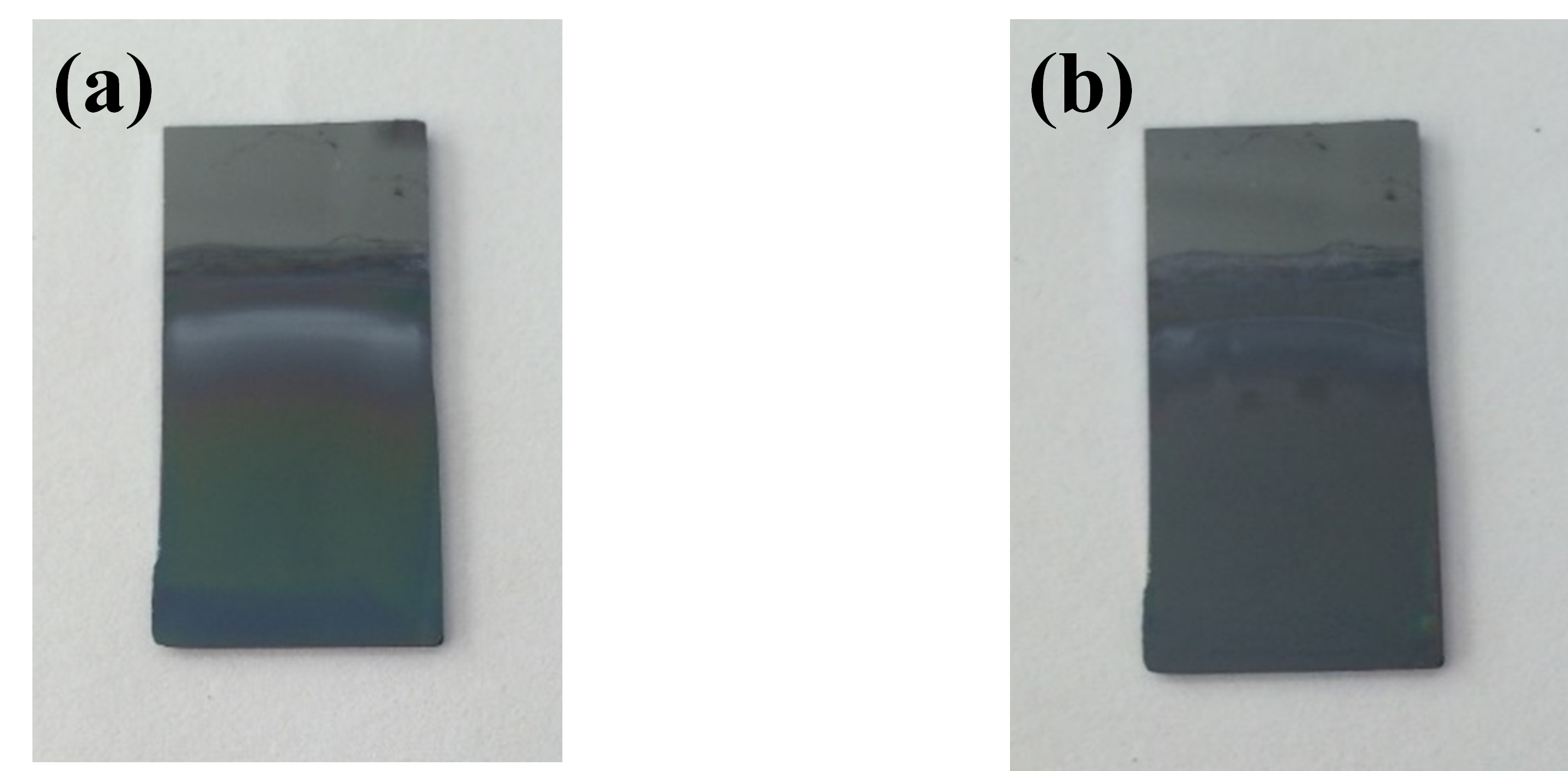


Images of BF films, before (a) and after (b) one step of the tape-peeling test

**Figure S7. The thickness change of a BF film during 3 h of water stability testing**

**
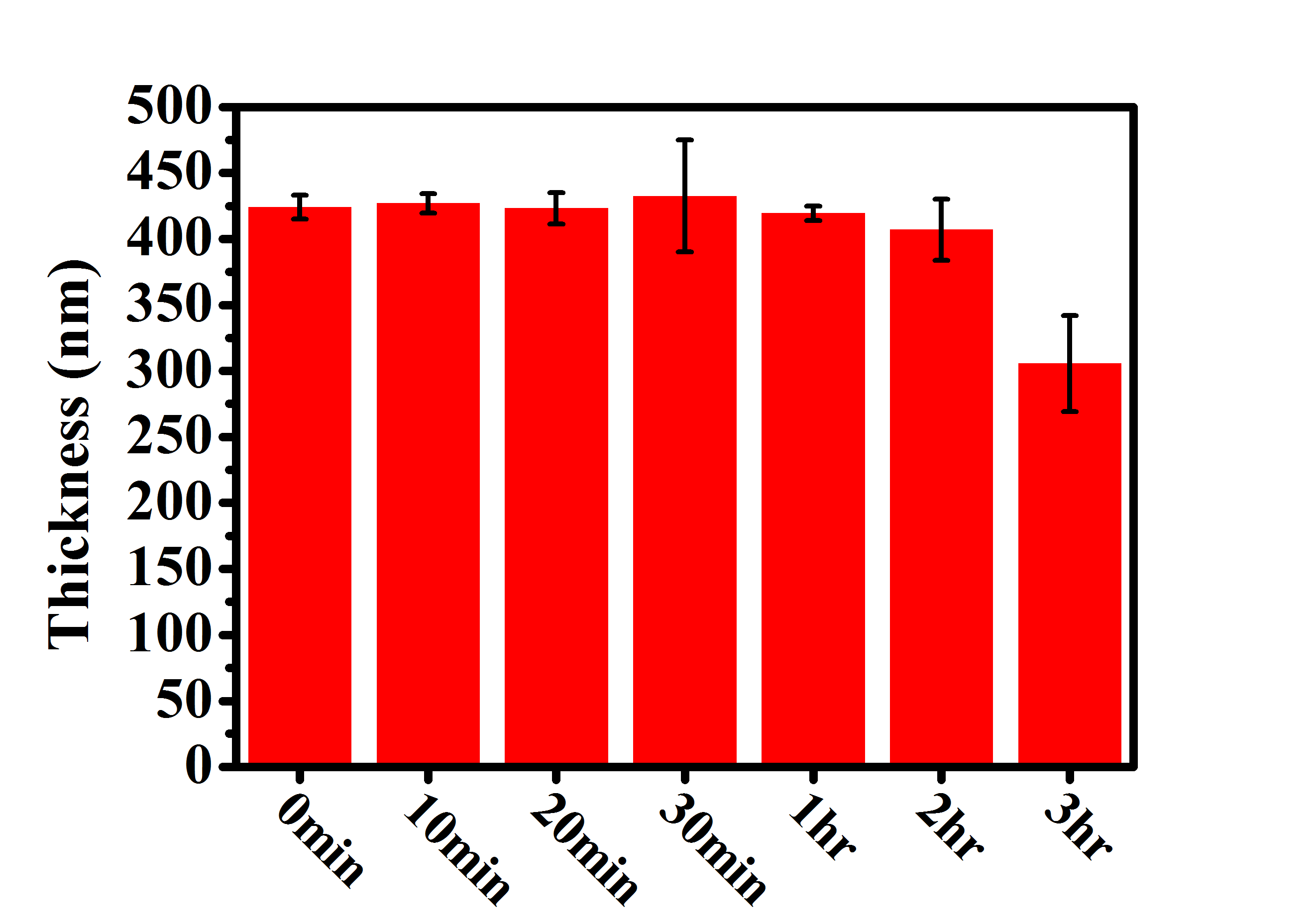
**

**Figure S8. Images of BFFA films after heat treatment**

**
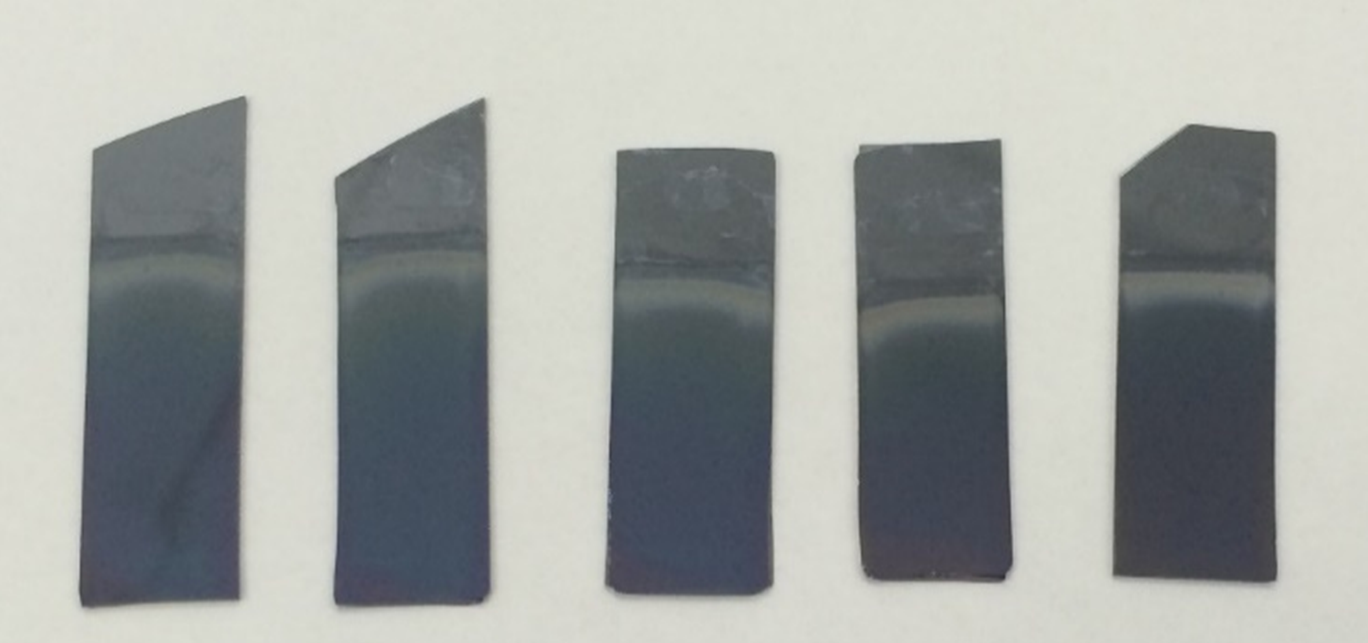
**

Images of BFFA films after heat treatment for 1 h at temperatures of 100, 200, 300, 400, and 500 °C (from left to right).

**Figure S9. Dynamic drop experiment of BFFA film**Movie file attached
